# Reconstruction of a Right Scapular Defect Using a Trapezius Flap After Necrotizing Fasciitis: A Case Report

**DOI:** 10.7759/cureus.96791

**Published:** 2025-11-13

**Authors:** Jesus Francisco Saltaren Fonseca, Valeria Rojas Ruiz, Juan N Daza Lopez, Andres C Herrera, Diego F Arango Ardila

**Affiliations:** 1 Plastic and Reconstructive Surgery, Hospital Occidente de Kennedy, Bogotá, COL; 2 Plastic Surgery, Hospital Occidente de Kennedy, Bogotá, COL

**Keywords:** chest wall reconstruction, myocutaneous flap, necrotizing fasciitis, scapular defect, trapezius muscle flap

## Abstract

Reconstruction of complex traumatic chest wall defects, particularly those complicated by necrotizing fasciitis (NF), requires a meticulous and individualized surgical approach. This case report describes the successful management of a 46-year-old male patient with post-traumatic NF involving the scapular region. After multiple surgical debridements and negative pressure wound therapy, definitive reconstruction was achieved using a chimeric trapezius muscle flap, providing both defect coverage and functional restoration. This case highlights the utility of the trapezius flap in managing challenging chest wall defects, particularly when alternative flap options are limited, and underscores the critical role of a multidisciplinary approach in the treatment of severe soft tissue infections.

## Introduction

Necrotizing soft tissue infections (NSTIs), including necrotizing fasciitis (NF), present a significant clinical challenge due to their rapid progression and high mortality rates [1,2]. These infections are characterized by necrosis of the skin, subcutaneous tissue, and fascia, and occur more frequently in patients with comorbidities such as diabetes mellitus and immunosuppression [3,4]. Prompt diagnosis, informed by clinical examination, laboratory data, and surgical findings, is essential [5,6]. While the Laboratory Risk Indicator for Necrotizing Fasciitis (LRINEC) score serves as a useful stratification tool, surgical exploration remains the definitive diagnostic method [3,6].

Management requires an aggressive, multidisciplinary approach combining broad-spectrum antimicrobial therapy with repeated surgical debridement [3,4]. Reconstruction of the resulting soft tissue defects, particularly in the chest wall, remains complex and technically demanding [7]. In this context, muscle flaps such as the trapezius have become valuable reconstructive options when traditional donor sites are unavailable or contraindicated [8,9].

Two primary types of trapezius flaps are described: those based on the superficial cervical artery (SCA), suitable for defects in the neck, mandible, and parotid regions; and those based on the dorsal scapular artery (DSA), which are more appropriate for reconstruction of the thoracic spine, scalp, and scapular areas. The choice of vascular pedicle and the orientation of the skin paddle determine the flap design [10]. In DSA-based flaps, the incision is typically made vertically between the scapular spine and the midline, allowing preservation of trapezius function during inset [9,11]. Flap selection and surgical technique must be tailored to the location and extent of the defect, as well as the availability of healthy adjacent tissues.

Successful outcomes depend on a comprehensive understanding of regional anatomy and precise intraoperative execution [9,11]. This case report describes the use of a chimeric trapezius flap for reconstruction in a patient with post-traumatic NF, highlighting its efficacy in addressing complex posterior thoracic defects.

## Case presentation

A 46-year-old male patient with no significant medical history presented with edema and pain in the right scapular region following a traumatic fall. Physical examination revealed localized edema, erythema, increased warmth, and a central indurated area measuring 15 × 10 cm. A chest radiograph demonstrated subcutaneous emphysema.

Based on the initial clinical impression of a soft tissue abscess, empirical antibiotic therapy was initiated with piperacillin-tazobactam and metronidazole. Laboratory evaluation showed an elevated C-reactive protein level (23.26 mg/dL) and leukocytosis (13,810/μL). Soft tissue ultrasonography revealed a 140 mL hypoechoic collection. Despite initial management, the patient developed progressive subcutaneous emphysema and functional limitation of the shoulder. Surgical drainage was performed, yielding approximately 1000 mL of purulent material. Microbiological cultures identified *Streptococcus viridans*.

**Figure 1:**
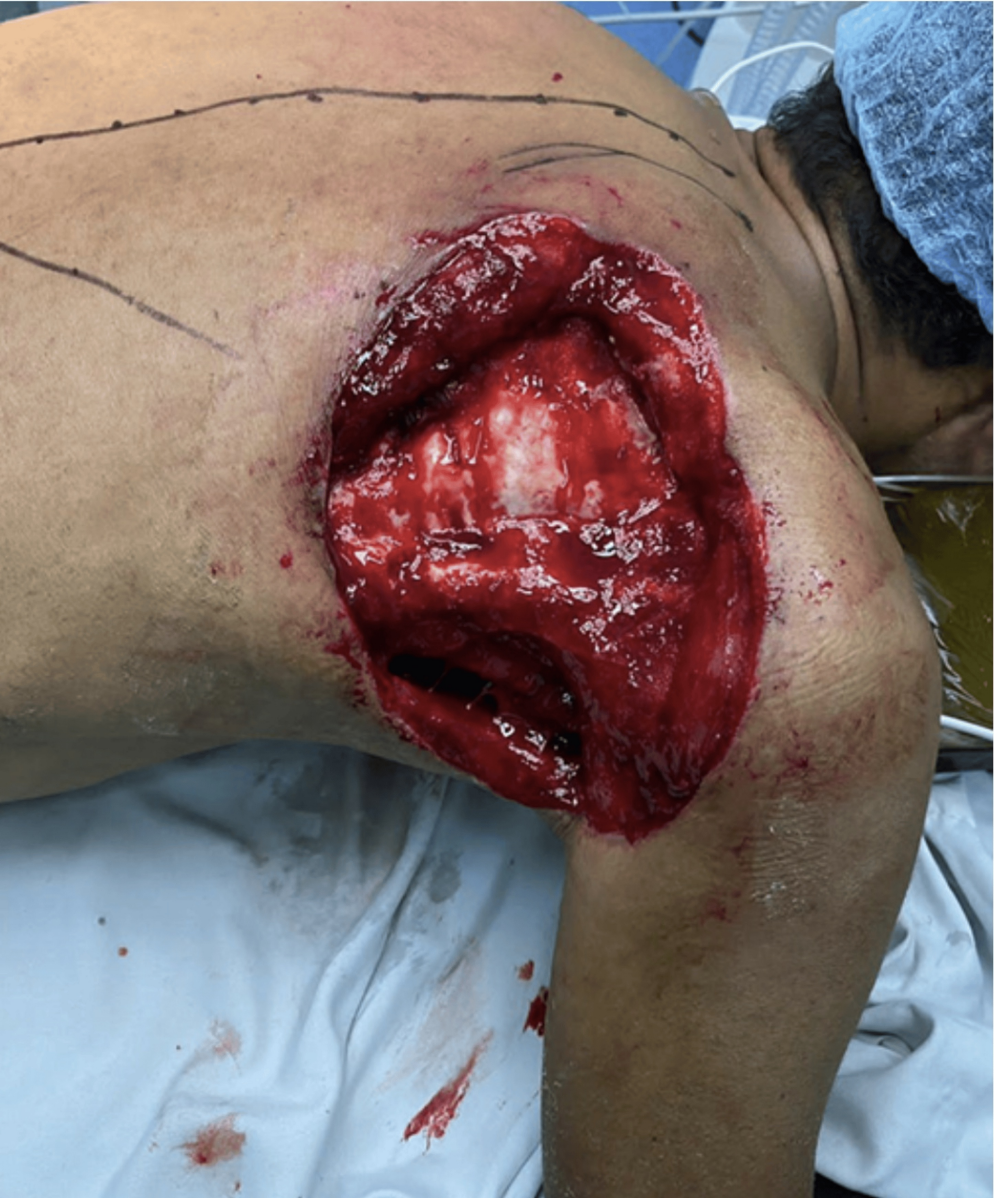
Soft tissue defect in the right scapular region following surgical debridement

A second surgical intervention was required due to increasing erythema and clinical deterioration. Intraoperative findings confirmed NF involving the scapular musculature, resulting in a soft tissue cavity measuring 20 × 20 cm (Figure 1). Negative pressure wound therapy was initiated postoperatively, and subsequent wound cultures were negative.

The plastic surgery team performed definitive reconstruction. Intraoperatively, there was a complete loss of the infraspinatus muscle and a partial absence of the supraspinatus, teres major, and teres minor muscles, resulting in significant impairment of posterior shoulder rotation.

**Figure 2:**
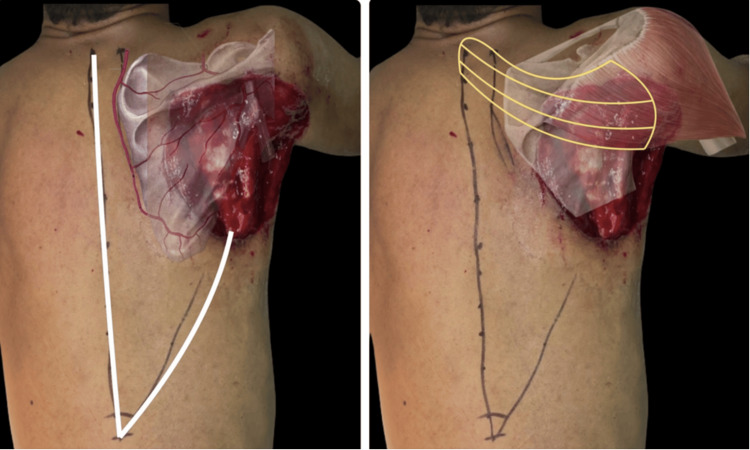
Intraoperative planning of the chimeric trapezius muscle flap based on the DSA DSA, dorsal scapular artery

To achieve defect coverage, a trapezius muscle flap was harvested from the right side, based on the DSA pedicle. The flap measured approximately 12 cm at its base and was rotated to provide complete coverage of the scapular defect. Fasciocutaneous dissection of the trapezius muscle was performed to elevate the muscular flap. The flap was anchored to the posterior belly of the deltoid muscle, the superior border of the latissimus dorsi, and the residual rhomboid muscle, aiming to restore shoulder function and enable retroversion (Figure 2).

**Figure 3:**
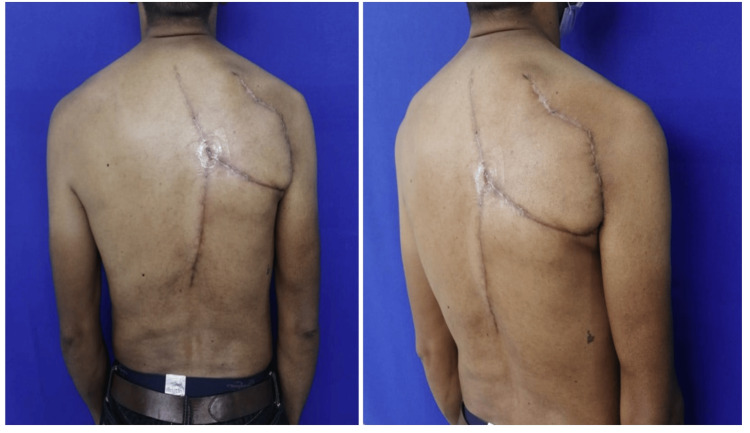
Flap evolution and shoulder mobility at the second postoperative month

Reconstruction with the trapezius flap resulted in full flap viability and complete coverage of the scapular defect. At discharge, functional recovery was evident, particularly in shoulder retroversion, which had previously been compromised. The postoperative course was favorable, with progressive improvement in shoulder mobility. At follow-up, the patient demonstrated active retroversion in response to elevation stimuli (Figure 3). 

## Discussion

NSTIs, such as NF, constitute surgical emergencies associated with high morbidity and mortality rates, ranging from 6% to 67% [1-3]. Common predisposing factors include diabetes mellitus, immunosuppression, and trauma [3,4,6]. Diagnosis relies primarily on clinical suspicion, particularly in the presence of hallmark symptoms such as erythema, edema, disproportionate pain, and subcutaneous emphysema.

Adjunctive diagnostic tools include the LRINEC score and imaging studies [3,6]. Computed tomography is considered the imaging modality of choice, offering high sensitivity (~100%) and specificity (80-98%) in detecting fascial thickening, edema, and gas within soft tissues [4,5]. Ultrasonography may assist in the early identification of fluid collections [4]. The “finger test,” involving a 2-cm incision down to the deep fascia, allows for visual and tactile assessment of necrosis, loss of resistance, and the presence of malodorous or “dishwater-like” fluid [2,6].

Following diagnosis, early administration of broad-spectrum antibiotics is imperative. Empirical regimens typically include vancomycin or linezolid in combination with piperacillin-tazobactam or a carbapenem, along with clindamycin to inhibit toxin production [3,5]. Despite optimized medical management, surgical debridement remains the cornerstone of treatment. However, reconstruction of the resultant soft tissue defects often poses a significant technical challenge [7].

Reconstructive options should be selected based on the location, size, and complexity of the defect [12]. In cases involving extensive soft tissue loss of the upper thoracic, scapular, or posterior cervical regions, the trapezius muscle flap serves as a reliable alternative, particularly when the pectoralis major or latissimus dorsi flaps are contraindicated or unavailable [9-11].

The trapezius muscle originates from the superior nuchal line, nuchal ligament, and spinous processes of C7-T12, inserting into the clavicle, acromion, and scapular spine. It is innervated by the spinal accessory nerve and cervical nerves C2-C4 [7]. The vascular supply is segmental: the upper third is supplied by the occipital artery, the middle third by the SCA, and the lower third by the DSA.

According to the dominant vascular pedicle, trapezius flaps are classified as SCA-based flaps, indicated for defects of the neck, mandible, and parotid regions, and DSA-based flaps, used for reconstruction of the thoracic spine, scalp, and scapular regions [10]. Flap designs may be myocutaneous or osteomyocutaneous, depending on the reconstructive goals [10]. Elevation techniques vary but typically involve incisions at the neck base or scapular border, with careful dissection to preserve the vascular pedicle and spinal accessory nerve [8,11].

In the present case, the scapular defect involved exposed bone and necessitated coverage with a DSA-based trapezius muscle flap. A vertically oriented island flap was designed to ensure sufficient vascular supply and optimal coverage. The surgical plan prioritized functional restoration by anchoring the flap to adjacent structures (deltoid, latissimus dorsi, and rhomboid muscles). The procedure was executed successfully, and the patient experienced a favorable recovery with restoration of shoulder movement.

Meticulous surgical planning, precise dissection, and intraoperative adaptability were essential for a successful outcome. Additionally, adherence to postoperative rehabilitation and coordination with a multidisciplinary team were critical in achieving long-term functional restoration.

## Conclusions

NF requires timely diagnosis and aggressive management, including broad-spectrum antibiotic therapy, surgical debridement, and hemodynamic stabilization. Early detection and prompt intervention are critical to improving patient outcomes. In cases involving extensive soft tissue loss, the trapezius flap represents a reliable and versatile reconstructive option for chest wall defects resulting from necrotizing infections. Its design and vascular supply allow for adaptation to various anatomical regions depending on the selected pedicle. Successful reconstruction demands careful planning of flap design, location, and vascular anatomy. In this case, the trapezius flap achieved effective defect coverage and contributed to functional restoration of the upper limb, ultimately preserving the patient’s quality of life.
